# Supplementary material for: Anaemia and Congestion in Heart Failure: Correlations and Prognostic Role
Source: Biomedicines. 2023 Mar 21;11(3):972. doi: 10.3390/biomedicines11030972 (PMC10046168; doi:10.3390/biomedicines11030972)
Supplement: Supplementary file 1 [file biomedicines-11-00972-s001.zip › biomedicines-2277575-supplementary.pdf]

Supplementary Materials

Anaemia and Congestion in Heart Failure: Correlations and Prognostic Role

Pietro Scicchitano, Massimo Iacoviello, Antonio Massari, Micaela De Palo, Angela Potenza, Raffaella Landriscina, Silvia Abruzzese, Maria Tangorra, Piero Guida, Marco Matteo Ciccone, Pasquale Caldarola and Francesco Massari

Table S1. Comparison between AHF and CHF patients of the main differences in term of bioimpendace analysis, estimated creatinine clearance and brain natriuretic peptide.

	All	AHF	CHF
Number of patients	434	182	252
BIVA, hydration index, %	76 ± 5.5	78±5.5	74±3.4
eCrCl, ml/min per 1.73 m^2	57 ± 29	47±25	64±29
BNP, pg/ml, median (95% CIs)	516 (423-582)	1006 (859-1247)	258 (218-302)

Abbreviations: AHF: acute heart failure; BIVA: bioimpedance vector analysis; BNP: brain natriuretic peptide; CHF: chronic heart failure; eCrCl: estimated creatinine clearance.
